# Association of birth weight with abdominal obesity and weight disorders in children and adolescents: the weight disorder survey of the CASPIAN-IV Study

**DOI:** 10.15171/jcvtr.2017.24

**Published:** 2017-08-21

**Authors:** Hossein Ansari, Mostafa Qorbani, Fatemeh Rezaei, Shirin Djalalinia, Mojgan Asadi, Sareh Miranzadeh, Mohammad Esmaeil Motlagh, Sahel Bayat, Saeid Safiri, Omid Safari, Morteza Shamsizadeh, Roya Kelishadi

**Affiliations:** ^1^Department of Epidemiology and Biostatistics, Health Promotion Research Center, Zahedan University of Medical Sciences, Zahedan, Iran; ^2^Non-communicable Diseases Research Center, Alborz University of Medical Sciences, Karaj, Iran; ^3^Chronic Diseases Research Center, Endocrinology and Metabolism Population Sciences Institute, Tehran University of Medical Sciences, Tehran, Iran; ^4^Department of Social Medicine, Medical School, Jahrom University of Medical Sciences, Jahrom, Iran; ^5^Development of Research & Technology Center, Deputy of Research and Technology, Ministry of Health and Medical Education, Tehran, Iran; ^6^Osteoporosis Research Center, Endocrinology and Metabolism Clinical Sciences Institute, Tehran University of Medical Sciences, Tehran, Iran; ^7^Pediatrics Department, Child Growth and Development Research Center, Research Institute for Primordial Prevention of Non Communicable Disease, Isfahan University of Medical Sciences, Isfahan, Iran; ^8^Department of Pediatrics, Ahvaz Jundishapur University of Medical Sciences, Ahvaz, Iran; ^9^Managerial Epidemiology Research Center, Department of Public Health, School of Nursing and Midwifery, Maragheh University of Medical Sciences, Maragheh, Iran; ^10^Department of Medical Surgical Nursing, School of Nursing and Midwifery, Hamadan University of Medical Sciences, Hamadan, Iran

**Keywords:** Birth Weight, Obesity, Overweight, Underweight, Children, Adolescents

## Abstract

***Introduction:*** This study aims to evaluate the association of birth weight (BW) with weight disorders in a national sample of Iranian pediatric population.

***Methods:*** This nationwide survey was conducted among 25000 student’s aged 6-18 year-old students, who were selected using multistage cluster random sampling from 30 provinces of Iran in 2011-2012. Anthropometric measures were measured under standard protocols by using calibrated instruments. Abdominal obesity was defined based on waist circumference (WC) ≥90th percentile value for age and sex. The WHO criterion was used to categorize BMI. Students’ BW was asked from parents using validate questionnaire and was categorized as low BW (LBW) (BW <2500 g), normal BW (NBW) (BW: 2500-4000 g) and high BW (HBW) (BW>4000 g).

***Results:*** This national survey was conducted among 23043 school students (participation rate: 92.6%). The mean age of participants (50.8% boys) was 12.54 ± 3.31 years. Results of multivariate logistic regression show that LBW increased odds of underweight (OR [odds ratio]: 1.61; 95% CI: 1.37, 1.89) and students with HBW had decreased odds of underweight (OR: 0.74; 95% CI: 0.58, 0.93) compared to students with NBW. Students with LBW compared to student with NBW had decreased odds of overweight (OR: 0.83; 95% CI: 0.69, 0.98) and general obesity (OR: 0.73; 95% CI: 0.56, 0.95). On the other hand, HBW increased odd of overweight (OR: 1.28; 95% CI: 1.09, 1.50), generalized obesity (OR: 1.59; 95% CI: 1.29, 1.96) and abdominal obesity (OR: 1.29; 95% CI: 1.11, 1.49) compared to NBW group.

***Conclusion:*** BW is a determinant of weight disorders and abdominal obesity in childhood and adolescence. This finding underscores the importance of prenatal care as well as close monitoring of the growth pattern of children born with low or high BW.

## Introduction

Underweight and overweight are both important health indicators in the pediatric age group. Most developing countries are facing a dual burden of nutritional disorders. While underweight and micronutrient deficiency still persist, overweight and obesity had rapid escalating trend.^1^ In 2010, 43 million children were estimated to be overweight and obese, and 92 million children were at risk of overweight.^2^

The worldwide prevalence of overweight and obesity in childhood has increased from 4.2% in 1990 to 6.7% in 2010. The prevalence is expected to reach 9.1% in 2020,^2,3^ with a considerably high prevalence in developing countries.^4^

Iran is a middle-income country that experienced a rapid epidemiological transition,^5^ in which a high prevalence of blood pressure, obesity, and type 2 diabetes has been demonstrated.^6^ Now, obesity is the most common nutritional disorder in Iranian children and adolescents.^7^ Overweight and obesity is a major public health problem among Iranian children.^8^ In a study that was conducted in Iran, the prevalence of overweight and obesity in 6-18 year-old children was 10.1% and 4.79%, according to national cut-off points.^9^ In a similar study, the prevalence of overweight and obesity was 12.67% and 10.47% in 7-18-year-old children.^10^

Obesity is a multifactorial disorder in which genetic, socio-economic status (SES), lifestyle, physical activity, and eating habits are of important predisposing factors.^11^ Overweight and obesity in adolescents are risk factors for non-communicable disease and higher mortality rates in adulthood.^12-14^

Positive association between birth weight (BW) and body mass index (BMI) in school-aged children and adolescents has been shown in a number of populations.^15,16^ Often, children who are overweight at the age of 5-7 years had high BW.^17^

Previous studies on the relationship between BW and BMI in childhood showed conflicting results. Therefore, a U-shaped relationship is suggested, i.e. children born with low BW (LBW) (<2500 g)^18^ or high BW (HBW) (>4000 g)^19^ both are at risk of excess weight. Positive association between high BW and childhood obesity has been shown in some studies,^20,21^ but not confirmed in another study.^22^ Limited experience exists in this regard in the Middle East and North Africa (MENA) region. This study aims to determine the association of BW with abdominal obesity and weight disorders in Iranian children and adolescents.

## Materials and Methods

The CASPIAN-IV study was performed in 2011-2012 in urban and rural areas of 30 provinces in Iran^23^ regarding the importance and priority of problem, a complementary part of this national survey focused on weight disorders evaluation.^24,25^

Through this investigation, 23183 school students aged 10-18 years were randomly selected and following the WHO-global school-based student health survey (GSHS-WHO) protocols, trained research experts involved in processes of examinations and inquiry. Information was recorded in the checklists and validated questionnaires for all participants.^26^

After explanation of the study, participants and one of their parents were assured that their responses would remain anonymous and confidential. Participation in the study was voluntary and all of potential participants had the right to withdraw from the study at any time. Oral assent and written informed consent were obtained from students and one of their parents respectively.

Aim to assess the standards of coordination and quality of data, all levels of quality assurance were closely supervised and monitored by Data and Safety Monitoring Board (DSMB) of the project.^23,26,27^

### Definition of Terms

Demographic information: Demographic information including age, sex, residence area, family characteristics, family history (FH) of obesity, parental level of education, possessing a family private car, type of home etc. Some complementary information on screen time, physical activity, and many other components of life styles were also questioned.

Birth weight: Participants’ birth weight (BW; g) was asked from their parents verbally and then categorized into three groups; LBW (BW <2500 g), normal BW (NBW) (BW: 2500-4000 g), and HBW (BW >4000 g) for statistical analysis.

SES: SES of families was estimated under based on the Progress in the International Reading Literacy Study (PIRLS) for Iran.^28^ Using principle component analysis (PCA), variables of parents’ education, parents’ job, possessing private car, school type (public/private), type of home (private/rented) and having personal computer in home were combined as one main component of SES.^29,30^ This estimated scale was categorized into 5 quintiles through which, the first quintile was defined as a “lowest SES”, and the fifth quintile as a “highest SES” groups.

Screen time (ST): The sum of the average daily hours spent for watching television or video, as well as for leisure time use of personal computer (PC) or electronic games (EG) was considered as ST. ST was asked separately for week days and weekends. For the analysis of correlates of ST, according to the international ST recommendations, ST was categorized into two groups; less than 2 hours per day (low), and 2 hours per day or more (high).^31^

Physical activity (PA): The recalls of physical activities in the prior week to the study were collected. Participants reported the weekly frequency of their leisure time PA outside the school. PA considered as at least 30 minutes duration of exercises per day which was led to heavy sweating or large increases in breathing or heart rate. PAQ-A instrument used to PA categorizing ; low physical activity level, that included those who scored between 1 to 1.9 on the PAQ-A instrument and moderate and high physical activity level that included participants with estimated scores between 2-5 PAQ-A (moderate level of 2 to 3.9 scores and high level with range of 4 to 5 score).^32^

### Measurements

A team of trained health care experts performed the examinations under standard protocols by using calibrated instruments. Weight was measured in light clothing to the nearest 100 g, and height without shoes to the nearest 10 cm while the students were standing and the shoulders and in normal position. BMI was calculated as weight (kg) divided by height squared (m^2^). Waist circumference (WC) was measured by a non- elastic tape to the nearest 0.1 cm at the end of expiration at the midpoint between the top of iliac crest and the lowest rib in standing position.^23^ Abdominal obesity was defined based on WC ≥90th percentile value for age and sex. The WHO criteria was used to categorized BMI; underweight was considered as <5th percentile, normal as 5th–84th percentile, overweigh as 85th–94th percentile, and obese as >95th percentile.^33,34^

### Statistical analysis

Categorical variables presented as a percentage with 95% CI. Mean of continuous variables shown with 95% CI. Comparisons of continuous and categorical variables across BW and anthropometric measures categories were assessed by using analysis of variance (ANOVA) and Pearson chi-square tests. To evaluate the association between BW duration and anthropometric measurements in different models, adjusting was done for possible confounders. Model I is a crude model (without adjustment) Logistic regression analyses were used. In Model II, the association was Adjusted for age, sex and living area. In Model III family history of obesity, socioeconomic status, physical activity, screen time, birth order, birth order, type of complementary feeding, Type of milk consumed and breastfeeding duration were analyzed using survey data analysis methods using Stata software (release 12, Stata Corp, College Station); *P *< 0.05 was considered as statistically significant.

## Results

This national survey was conducted among 23 043 school students (participation rate: 92.6). The mean age of participants was 12.54 ± 3.31 years. Totally, 50.8% of students were boys and 73.5% resided in urban areas. The characteristics of the study participants by BW categories are presented in Table 1. Distribution of sex, living area, ST, family size, type of complementary feeding, type of milk consumed and duration of breastfeeding was different according to categories of BW (*P *< 0.05).

**Table 1:** Characteristics of participants according to birth weight categories: the weight disorders survey of the CASPIAN-IV study

	**BW categories (g)**				**P value**
	**Total**	**<2500**	**2500-4000**	**>4000**	
Age (y)^2^	12.53 [12.48,12.57]	12.58 [12.44, 12.72]	12.50[12.45,12.54]	12.63 [12.48,12.79]	0.14
Living place^2^					
Urban	73.43[70.89,75.82]	70.57[66.73,74.14]	73.48[77.53,83.99]	80.97[71.25,76.16]	<0.001
Rural	26.57[24.18,29.11]	29.43 [25.86,33.27]	26.52[24.09,29.1]	19.03[16.01,22.47]	
Sex^2^					
Boy	50.84 [48.11,53.56]	47.47[43.77,51.2]	50.80[48.04,53.55]	55.43[51.2,59.58]	0.0012
Girls	49.16[46.44,51.89]	52.53[48.8,56.23]	49.20[46.45,51.96]	44.57 [40.42,48.8]	
Family history of obesity^2^					
Yes	24[23.27,24.74]	22.96[21.11,24.93]	24.16[23.4,24.93]	23.56[21.41,25.86]	0.46
No	76[75.26,76.73]	77.04[75.07,78.89]	75.84[75.07,76.6]	76.44[74.14,78.59]	
Birth order^2^					
1st	44.88 [44.06,45.7]	43.35[41.03,45.7]	45.32[44.42,46.21]	42.02[39.49,44.59]	0.11
2nd	31[30.32,31.69]	31.66[29.52,33.88]	30.86[30.13,31.6]	31.67[29.35,34.09]	
3rd	16.08 [15.51,16.66]	16.12[14.51,17.88]	15.94[15.33,16.57]	17.52[15.61,19.61]	
>4th	8.04[7.61,8.49]	8.86[7.65,10.24]	7.88[7.42,8.36]	8.79[7.48,10.31]	
Family size^2^					
≤4 person	83.71[82.82,84.57]	80.50 [78.39,82.45]	83.92 [82.99,84.8]	85.49[83.42,87.34]	<0.001
>4 person	16.29[15.43,17.18]	19.50[17.55,21.61]	16.08[15.2,17.01]	14.51[12.66,16.58]	
Screen time^2^					
≤ 2 h/d	59[57.96,60.02]	62.02[59.65,64.33]	58.82[57.73,59.9]	57.22[54.56,59.84]	0.01
>2 h/d	41[39.98,42.04]	37.98 [35.67,40.35]	41.18[40.1,42.27]	42.78[40.16,45.44]	
Physical activity^2^					
Active	76.56[75.33,77.74]	75.16[72.85,77.34]	76.56[75.3,77.77]	78.29[75.93,80.48]	0.10
Inactive	23.44[22.26,24.67]	24.84[22.66,27.15]	23.44[22.23,24.7]	21.71[19.52,24.07]	
Socio-economic status^2^					
1	19.75[18.71,20.83]	26.80 [24.3,29.47]	19.43[18.35,20.56]	14.03[12.14,16.16]	<0.001
2	19.08[19.02,20.6]	21.70 [19.84,23.69]	19.69[18.87,20.54]	18.43[16.34,20.72]	
3	20.18 [19.45,20.93]	20.84 [18.82,23.01]	20.01[19.25,20.80]	21.19[19.11,23.43]	
4	20.09[19.31,20.9]	15.95[14.26,17.80]	20.35[19.52,21.22]	22.61[20.39,24.99]	
5	20.18 [18.91,21.52]	14.07[12.88,16.74]	20.51[19.19,21.89]	23.74[21.10,26.61]	
Type of complementary feeding^2^					
Always homemade foods	73.55[72.47,74.61]	73.87[71.67,75.95]	73.96[72.82,75.06]	68.83 [66.36,71.2]	<0.001
Always formula	2.47 [2.22,2.74]	4.00 [3.19,5.02]	2.31[2.07,2.58]	2.28[1.65,3.13]	
Usually homemade foods	21.05[20.13,22.01]	18.49[16.67,20.46]	20.96[19.98,21.97]	25.26[23.13,27.52]	
Usually formula	2.92[2.64,3.23]	3.63 [2.88,4.57]	2.77[2.47,3.10]	3.62[2.812,4.664]	
Type of milk consumed^2^					
Breast feeding	81.60[80.89,82.28]	68.76[66.46,70.98]	82.88[82.12,83.61]	83.60[81.57,85.44]	<0.001
Formula	3.72[3.46,4.01]	8.23[7.07,9.57]	3.27[3.00,3.56]	3.08[2.37,3.99]	
Cow’s milk	5.72[5.29,6.20]	9.22[7.89,10.76]	5.49[5.03,6.00]	3.89[3.04,4.97]	
Mixed	8.94[8.44,9.47]	13.77[12.26,15.44]	8.34[7.82,8.90]	9.24[7.96,11.12]	
Breastfeeding duration (month)^1^	16.36 [16.24, 16.48]	14.48 [14.04,14.93]	16.58[16.45,16.71]	16.24 [15.75,16.73]	<0.001

^1^are presented as mean (95%CI) ^2^are presented as percentage (95%CI)

Table 2 shows mean (95% CI) of anthropometric measures according to BW categories. A significant association was found between BW categories and all anthropometric measures except wrist circumference (*P *< 0.05). Participants HBW had significantly higher mean (95% CI) of weight (46.27 [44.8, 47.73] kg), height (149.68 [148.18, 151.18] cm), BMI (19.80 [19.50, 20.11] kg/m^2^), WC (69.37 [68.50, 70.24] cm), hip (83.68 [82.62, 84.75] cm], neck (30.98 [30.65, 31.32] cm) compared to other groups.

**Table 2:** Mean (95% CI) of anthropometric measures according to birth weight categories: the weight disorders survey of the CASPIAN-IV study

	**BW categories (g)**			
	**<2500**	**2500-4000**	**>4000**	***P *** **value**
Weight (kg)	40.02 (38.96, 41.08)	42.45 (41.65,43.26)	46.27 (44.8,47.73)	<0.001
Height (cm)	145.82 (144.53,147.11)	147.33 (146.38,148.28)	149.68 (148.18,151.18)	<0.001
BMI (kg/m^2^)	18.14 (17.88,18.40)	18.80 (18.65,18.95)	19.80 (19.50,20.11)	<0.001
WC (cm)	65.01 (64.32,65.70)	66.76 (66.30,67.22)	69.37 (68.50,70.24)	<0.001
Wrist (cm)	15.46 (14.16,16.76)	15.37 (15.09,15.64)	15.62 (15.24,15.99)	0.86
Hip (cm)	79.43 (78.52,80.33)	80.95 (80.33,81.57)	83.68 (82.62,84.75)	<0.001
Neck (cm)	29.75 (29.49,30.01)	30.38 (30.19,30.57)	30.98 (30.65,31.32)	<0.001

Abbreviations: BMI, body mass index; WC, waist circumference.

**Figure 1:**
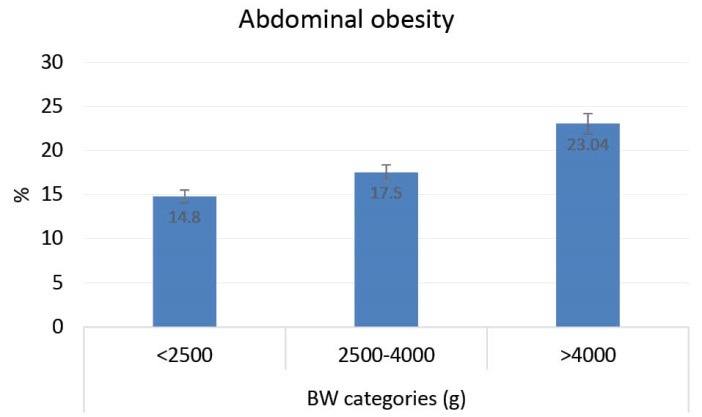
Prevalence of abdominal obesity according to birth weight categories: the weight disorders survey of the CASPIAN IV study.

**Figure 2:**
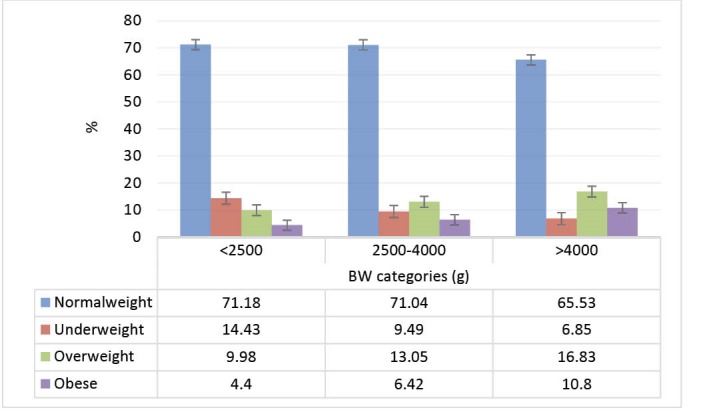
Prevalence of weight disorders according to birth weight categories: the weight disorders survey of the CASPIAN IV study.

Prevalence of abdominal obesity and weight disorders according to BW categories is presented in Figures 1 and 2 respectively. Prevalence of overweight, generalized obesity and abdominal obesity increase linearly by increasing BW (*P *< 0.001). Prevalence of underweight linearly decreased per BW increment (*P *< 0.001).

Association of BW with weight disorders and abdominal obesity in logistic regression analysis is presented in Table 3. In the multivariate model (model III), LBW increased odds of underweight (OR [odds ratio]: 1.61; 95% CI: 1.37, 1.89) and students with HBW had decreased odds of underweight (OR: 0.74; 95% CI: 0.58, 0.93) compared to students with NBW. Students with LBW compared to student with NBW had decreased odds of overweight (OR: 0.83; 95% CI: 0.69, 0.98) and general obesity (OR: 0.73; 95% CI: 0.56, 0.95). On the other hand, HBW increased odd of overweight (OR: 1.28; 95% CI: 1.09, 1.50), generalized obesity (OR: 1.59; 95% CI: 1.29, 1.96) and abdominal obesity (OR: 1.29; 95% CI: 1.11, 1.49) compared to NBW group.

**Table 3:** Odds ratios (95% CI) for weight disorders and abdominal obesity according to birth weight categories: the weight disorders survey of the CASPIAN-IV study

	**BW categories (g)**		
	**2500-4000**	**<2500**	**>4000**
Underweight			
Model I	Ref.	1.60(1.40,1.84)*	0.7(0.57,0.85)*
Model II	Ref	1.58(1.38,1.81)*	0.72(0.59,0.87)*
Model III	Ref	1.61(1.37,1.89)*	0.74(0.58,0.93)*
Overweight			
Model I	Ref	0.73(0.63,0.85)*	1.34(1.18,1.53)*
Model II	Ref	0.75(0.64,0.86)*	1.30(1.14,1.49)*
Model III	Ref	0.83(0.69,0.98)*	1.28(1.09,1.50)*
General obesity			
Model I	Ref	0.67(0.53,0.84)*	1.76(1.49,2.08)*
Model II	Ref	0.68(0.54,0.86)*	1.71(1.44,2.02)*
Model III	Ref	0.73(0.56,0.95)*	1.59(1.29,1.96)*
Abdominal adiposity			
Model I	Ref	0.81(0.71,0.93)*	1.40(1.24,1.58)*
Model II	Ref	0.84(0.73,0.96)*	1.34(1.19,1.52)*
Model III	Ref	0.89(0.77,1.04)	1.29(1.11,1.49)*

Model I: crude model; Model II: adjusted for age, sex and living area; Model III: additionally adjusted for family history of obesity, socioeconomic status, physical activity, screen time, birth order, Type of complementary feeding, Type of milk consumed and breastfeeding duration. *Statistically significant

Table 4 represents association of BW with anthropometric measures in linear regression model. In the multivariate model (model III), student with LBW had lower BMI (-β: 0.48; 95% CI: -0.73,-0.23), neck (β: -0.57; 95% CI: -0.78,-0.37), WC (β:-1.21; 95% CI: -1.72,-0.70) and hip (β: -1.37; 95% CI: -1.90,-0.83) compared to students with NBW. Also student with HBW had significantly higher BMI, neck, WC and hip compared to students with NBW. Association of wrist with anthropometric measures was not statistically significant.

**Table 4:** Beta coefficients (95% CI) for anthropometric measures according to birth weight: the weight disorders survey of the CASPIAN-IV study

	**BW categories (g)**		
	**2500-4000**	**<2500**	**>4000**
BMI (kg/m^2^)			
Model I	Ref.	-0.66(-0.89,0.44)*	1.00(0.72,1.27)*
Model II	Ref.	-0.69(-0.88,-0.50)*	0.87(0.65,1.09)*
Model III	Ref.	-0.48(-0.73,-0.23)*	0.90(0.58,1.22)*
WC (cm)			
Model I	Ref.	-1.74(-2.35,-1.14)*	2.61(1.84,3.38)*
Model II	Ref.	-1.71(-2.16,-1.26)*	2.02(1.43,2.60)*
Model III	Ref.	-1.21(-1.72,-0.70)*	1.73(1.05,2.40)*
Wrist (cm)			
Model I	Ref.	0.09(-1.22,1.41)	0.25(-0.20,0.70)
Model II	Ref.	0.08(-1.24,1.42)	0.16(-0.28,0.61)
Model III	Ref.	-0.45(-0.95,0.04)	0.37(-0.21,0.96)
Hip (cm)			
Model I	Ref.	-1.52(-2.27,-0.77)*	2.73(1.82,3.64)*
Model II	Ref.	-1.68(-2.15,-1.21)*	2.18(1.62,2.74)*
Model III	Ref.	-1.37(-1.90,-0.83)*	1.95(1.31,2.59)*
Neck (cm)			
Model I	Ref.	-0.63(-0.86,-0.40)*	0.60(0.29,0.90)*
Model II	Ref.	-0.64(-0.82,-0.47)*	0.40(0.15,0.65)*
Model III	Ref.	-0.57(-0.78,-0.37)*	0.39(0.08,0.70)*

Abbreviations: BMI, body mass index; WC, waist circumference. Model I :crude model; Model II : adjusted for age, sex and living area; Model III: additionally adjusted for family history of obesity, socioeconomic status, physical activity, screen time, birth order, Type of complementary feeding, Type of milk consumed and breastfeeding duration *Statistically significant.

## Discussion

Results of present study show that BW is associated with weight disorders and abdominal obesity. Participants with LBW compared with those with NBW were 1.61 times more likely to be underweight during childhood and adolescence. In addition, an inverse association was observed between LBW with overweight and general obesity, i.e. participants with LBW had 17% (OR: 0.83) and 27% (OR: 0.73) lower odds of overweight and general obesity respectively (compared to NBW group). We also found an inverse association between BW and BMI, WC, hip and neck circumferences. This suggests that students with history of LBW compared with those born with NBW have lower BMI, WC, and hip and neck circumference. These findings are discordant with previous studies which show that LBW increased risk of cardiometabolic risk factors in children and adolescents.^35,36^ Results of a systematic review highlight the important role of LBW in increasing the risk of cardiometabolic risk factors in adulthood and in later life. This systematic review concluded that rapid postneonatal catch-up growth of LBW neonates is more important factor than LBW alone in increasing risk of cardiometabolic risk factors.^37^

Moreover, our results show that HBW increased odd of overweight and generalized obesity compared to NBW group which was concordant with previous studies.^38-40^ We also found, a direct relationship existed between HBW with BMI and WC, as well as with, hip and neck circumferences. This shows that children and adolescents with history of HBW had higher BMI, WC, hip, and neck circumference than those born with NBW. Our finding also is concordant with other findings that shown HBW is associated with obesity in later life, and prenatal period is a critical period for the development of adiposity.^41^ Yuan et al found that the association of BW and BMI is J-shaped in Chinese pediatric population.^41^ The results of some other studies have documented the relationships of BW with anthropometric measures including BMI, WC, hip and wrist circumference.^40-44^

It should be considered that obesity and overweight are multifactorial phenomenon and in addition to the effect of BW on obesity, genes and environmental factors such as cultural and social mediated dietary habits, and reduced domestic and living work activities are also involved in the obesity pandemic.^45^

In present study the association of HBW with abdominal obesity was not statistically significant. Inconsistent with our findings, the results of Tian et al study in Chines adults’ population show that BW is an independent risk factor for abdominal adiposity. In fact, both LBW and HBW are known as risk factors for abdominal adiposity.^40^

The differences of body weight and anthropometric indices in the pediatric age group depend on BW and other factors including age, ethnicity, and maternal glucose tolerance during pregnancy, changes in body composition during puberty, diet and physical activity, as well as hormonal changes.^43,44^

The main limitation of this study is its cross-sectional nature. The other limitation is using parent-reported data for BW status which was susceptible to recall bias. The strength of this study, which might overcome its limitations, was its nationwide coverage and large sample size which increase the generalizability of results.

## Conclusion

Our findings serve as confirmatory evidence on the association of BW with weight disorders in childhood and adolescence. The role of prenatal care in health promotion of the future generation should be highlighted in health policy making. Growth monitoring of children born with low or high BW needs to be emphasized.

## Competing interests

None.

## Ethical approval

The ethics committees and other relevant national and provincial regulatory organizations approved the study.

## Acknowledgements

The authors are thankful of the large team working on this study and all participants in different provinces.
